# Assessing the Perioperative Capacity in the Republic of Zambia in Preparation for the First Revision of the National Surgical Obstetric and Anesthesia Plan (NSOAP): A Rapid Survey Method

**DOI:** 10.1002/wjs.12615

**Published:** 2025-05-06

**Authors:** Joshua Gazzetta, Emmanuel Malabo Makasa, Moses Mwale, Cyrus Phiri, Mwamba Josephine Chiteba Mulenga, Mutimba Bernard Mpabalwani, Lillian Mwape, Kennedy Lishimpi

**Affiliations:** ^1^ Department of Surgery University of Virginia and Centre for Surgical Healthcare Research Charlottesville Virginia USA; ^2^ Centre for Surgical Healthcare Research Department of Surgery University Teaching Hospital Lusaka Zambia; ^3^ World Health Organization Lusaka Zambia; ^4^ Republic of Zambia Ministry of Health The Permanent Secretary Lusaka Zambia

**Keywords:** assessment, capacity, NSOAP, perioperative

## Abstract

**Background:**

Recognizing unmet surgical needs in low‐ and middle‐income countries (LMICs) has led to worldwide initiatives to scale‐up surgical capacity. The Republic of Zambia is preparing for its first National Surgical Obstetric and Anesthesia Plan (NSOAP) revision and there is limited data on Zambia's surgical healthcare capacity. We aim to highlight Zambia's surgical healthcare capacity to inform the NSOAP revision and the method used for the rapid assessment.

**Methods:**

The Emergency and Essential Surgical (Perioperative) Healthcare Health Facility Assessment Tool (ZAMSAT) was fashioned. ZAMSAT survey responses were used to provide a cross‐sectional assessment of leveled healthcare facilities nationwide. The survey was distributed to 227 first‐, second‐, and third‐level hospitals under the leadership of Zambia's Operating Theater Nurses Interest Group and the MOH. The WhatsApp messenger application was used for survey distribution and collection.

**Results:**

Data from 116 leveled facilities in all 10 provinces were captured for a survey response rate of 51.1%. A mix of public, private, and mission/nonprofit hospitals was included. The uninterrupted supply of electricity, oxygen, and clean water was found in less than 40% of all facilities. The overall ability to perform caesarean deliveries was 89%, but the ability to perform a laparotomy or open fracture management was significantly lower at 71% and 37%, respectively. Both the WHO Surgical Safety Checklist and pulse‐oximetry use in the theater are consistently used more than 85% of the time. General doctors and anesthesia providers are responsible for the majority of intraoperative care in Zambia. Two‐percent of facilities are using electronic records as their only means of medical record keeping. The most common research being performed is observational. Although 7% of facilities report a dedicated budget line for perioperative care, only 30% report the budget is adequate. Forty‐percent of facilities have local committees dedicated to perioperative care and only 43% of facilities were aware of the NSOAP (2017–2021).

**Conclusion:**

ZAMSAT successfully quantified nationwide perioperative healthcare capacity data to inform the Republic of Zambia's first NSOAP revision. The assessment highlights important gaps in each NSOAP domain that may be addressed to advance perioperative healthcare in Zambia.

## 1 Background

Nine of 10 people in low‐ and middle‐income countries do not have access to basic surgical needs and sub‐Saharan Africa has one of the greatest needs for surgical capacity growth [1, 2, 3]. Recognizing unmet surgical needs has led to worldwide initiatives to reduce the burden of surgical disease. The Lancet Commission on Global Surgery (LCoGS) introduced the National Surgical Obstetric and Anesthesia Plan (NSOAP) framework as a template that countries can use to develop their national surgical healthcare policies to scale up perioperative care [1]. After the global efforts that culminated in the passing of the World Health Assembly Resolution WHA68.15 (2015) and decision WHA70.22 (2017), Zambia translated its political commitments into action by developing the world's first NSOAP (2017–2021) [4, 5]. This groundbreaking initiative aimed to outline Zambia's roadmap to improve safe, timely, and affordable surgical care for its 20 million people. The implementation of Zambia's first NSOAP did not proceed as planned for many reasons. First, the COVID‐19 pandemic, the change in government in 2021, and the new government's need to revise the expired National Health Strategic Plan (NHSP) caused delays. Second, the lack of resources and unrealistic objectives prevented the deployment of large‐scale, nationwide projects. To overcome this barrier, three district‐level hospitals near the capital city have been chosen for surgical capacity building for the revised NSOAP. These sites will be used to pilot the implementation process and will be used as a framework to scale‐up surgical capacity nationwide over time. Lastly, the first NSOAP's monitoring and evaluation (M + E) assessment was not completed due to competing priorities.

The World Health Organization (WHO) recommends assessing surgical capacity before surgical strengthening policy development [6]. The WHO tool for situational analysis to assess emergency and essential surgical care was first developed for this purpose in 2007 [7]. This tool has been modified by different groups to improve its usability and tailor it to different country‐specific needs. Although several tools have been used to measure surgical capacity in LMICs, there is evidence that many have failed to accurately reflect surgical health system outputs [8]. Several of the available assessment tools also require significant financial and human resources to complete hospital walkthroughs and data collection training which is not feasible for many LMICs. The last surgical capacity assessment done in Zambia was a cross‐sectional assessment using a modified WHO questionnaire in 2016. It captured data on 39 of the surgically active district hospitals [9]. This report was an excellent step toward defining Zambia's surgical capacity but does not include information on several of the NSOAP domains including information management, research, financing, and governance. Additionally, it does not include several infrastructure and service delivery markers needed for Zambia's policy revision. Due to the paucity of baseline data available in Zambia, a surgical capacity assessment was performed.

The Emergency and Essential Surgical (Perioperative) Healthcare Health Facility Assessment Tool (ZAMSAT) was fashioned to follow the health system building blocks of infrastructure, service delivery, workforce, information management, research, financing, and governance similar to the NSOAP framework generated by the LCoGS. ZAMSAT (Supporting Information S1) provides a simple scaffold for policy construction and allows for future use so that the successes and failures of the implementation process may be accurately measured in concert with the M + E assessments. Similar to the Surgeons OverSeas (SOS) personal, infrastructure, procedures, equipment, and supplies (PIPES) tool, which has been successfully used in many countries [10, 11, 12, 13, 14], ZAMSAT records binary answers for many of the indicators. ZAMSAT complements the NSOAP's M + E assessments by providing overall densities while the M + E assessments provide data on action points using defined indicators to measure specific implementation activities.

Following the pilot phase, the tool was used to capture nationwide health facility surgical healthcare capacity data. This research aims to (1) evaluate Zambia's surgical capacity and (2) highlight the rapid assessment method used to inform Zambia's NSOAP revision.

## 2 Methods

### 2.1 Setting

The Republic of Zambia is a landlocked country in Sub‐Saharan Africa with a population of approximately 20 million people. There are nearly 12 million people who live in rural communities and 8 million people who live in an urban setting. Zambia is 752,618 square kilometers with 72 ethnicities represented. The high fertility rate of 4.42 children born per woman drives rapid population growth, which has quadrupled since 1969. Life expectancy at birth is 61 years [15, 16]. According to the Zambia Harmonized Health Facility Assessment (ZHHFA), there are 40 third‐level hospitals (tertiary care hospitals), 33 second‐level hospitals (provincial hospitals), and 154 first‐level hospitals (district hospitals). Of these, approximately 55% have reported offering any type of surgical services [17, 18]. In Zambia, all leveled hospitals aim to provide surgical services including emergency cesarean sections, open fracture management, and laparotomies.

### 2.2 Rapid Assessment Approach

In 2024, the Center of Surgical Healthcare Research (CSHR) and the WHO–Zambia country office partnered with the Ministry of Health (MOH) to create a working group comprised of policy and surgical healthcare experts to plan the rapid assessment. The CSHR is a nongovernmental organization (NGO) hosted in the Department of Surgery at the University Teaching Hospital in Zambia's capital city, Lusaka. Further input was provided by key stakeholders, including national medical and surgical professional society leaders and Zambia's Operating Theater Nurses Interest Group (ZOTNIG). ZAMSAT was subsequently piloted at the University Teaching Hospital, Levi‐Mwanawasa Teaching Hospital, and the National Heart Hospital. Multiple iterations were performed during and after the piloting phase. ZAMSAT was approved by the MOH for use in assessing surgical healthcare capacity to inform the revision of Zambia's NSOAP.

ZAMSAT consists of seven sections created to align with Zambia's NSOAP formulation and modified from the NSOAP framework provided by the LCoGS. ZAMSAT data were administered by the perioperative nurses at each first‐, second‐, and third‐level facilities under the leadership of ZOTNIG from August 1, 2024 to August 30, 2024. All ZOTNIG members were given the survey electronically using the WhatsApp messaging platform, guided on administration by the lead working group members, and provided technical support during the collection period. Other stakeholders, including professional society leaders across specialties and the MOH, were engaged to encourage timely data collection. Data analysis was conducted using descriptive statistics and chi‐squared tests to identify key patterns and disparities across the healthcare system. Logistic regression was used to determine variable associations. Statistical analyses were performed using IBM SPSS Statistics with the R Integration Package. *p*‐values < 0.05 identified significance.

## 3 Results

**FIGURE 1:**
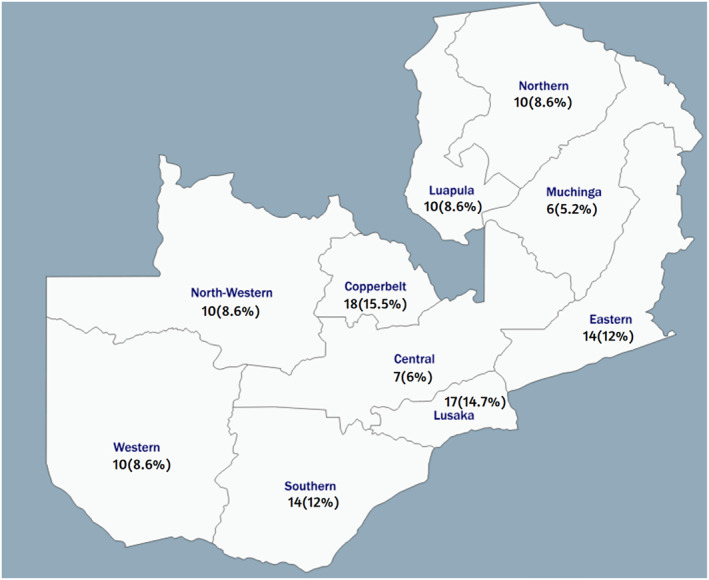
Distribution of facilities assessed.

**FIGURE 2:**
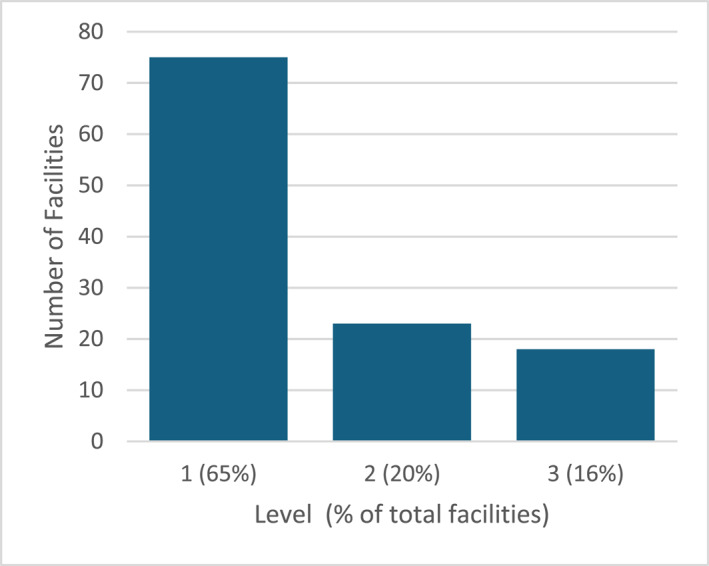
Facilities assessed by level.

**FIGURE 3:**
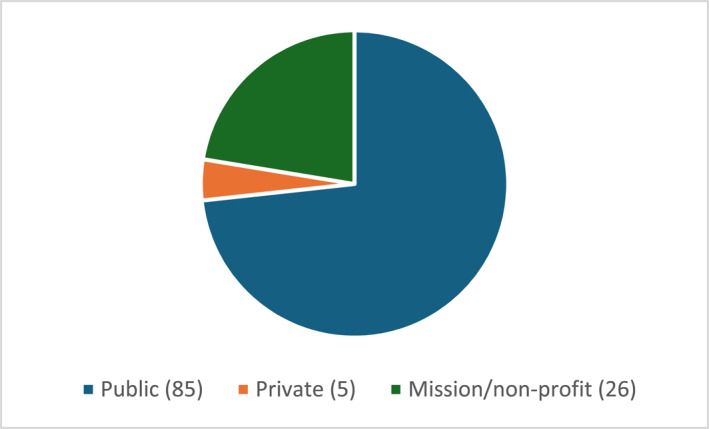
Facilities assessed by ownership.

Data were captured from 116 health facilities in all 10 provinces (Figure 1), representing 51.1% of all Level 1, 2, and 3 hospitals (Figure 2). A mix of public, private, and mission/nonprofit hospitals was included (Figure 3).

### 3.1 Infrastructure

Although consistent internet availability is relatively high across all facilities at 77%, critical resources such as electricity, clean water, oxygen, and ambulance services are only reliably available a minority of the time (Table 1). Private hospitals reported electricity interruptions at 20%, whereas mission/nonprofit and public facilities reported electricity interruptions at 85% and 70%, respectively. Compared to public facilities, private and mission/nonprofit facilities had fewer interruptions to perioperative care due to oxygen supply (OR 0.47, CI 95% 0.24–0.93, *p* = 0.032). Additionally, compared to public facilities, internet interruptions were more strongly associated with private and mission/nonprofit facilities (2.16, 1.01–4.46, *p* = 0.041). Seventy‐nine percent of facilities report that a lack of equipment and supplies interrupts surgical care.

**TABLE 1:** Overall infrastructure availability.

Infrastructure	Reliable availability (%)
Electricity	28.24
Clean water	37.04
Oxygen	37.96
Internet	77.31
Ambulance services	51.85

### 3.2 Service Delivery

The analysis of service delivery across health facilities reveals notable differences in the ability to perform Bellwether procedures, which include caesarean delivery, laparotomy, and treatment of open fractures. Per the LCoGS, Bellwether procedure capacity is a service delivery benchmark for which procedures all leveled hospitals should be able to perform 24/7 [1, 19]. As noted in Table 2, the ability to perform cesarean sections was 89%. In contrast, the ability to perform emergency laparotomies and manage open fractures was much lower at 71% and 37%, respectively. There is consistent use of pulse‐oximetry and the WHO Surgical Safety Checklist across most facilities.

**TABLE 2:** Service delivery.

Service availability	Percentage (%)
Emergency laparotomy availability	71.76
Caesarean section availability	89.35
Open fracture management availability	37.04
WHO surgical safety checklist usage	86.57
Pulse‐oximetry usage in operating rooms	96.76

### 3.3 Workforce

General doctors and anesthesia providers perform most of the perioperative theater care, with over 80% of facilities having these professionals available at all times (Table 3).

**TABLE 3:** Workforce providers.

Provider	24/7 availability (%)
General surgeon	57.87
Orthopedic surgeon	27.78
Anesthesia provider	86.57
OB/GYN	59.72
General doctor	82.41

### 3.4 Information Management

Fifty‐percent of facilities reported using only paper records, whereas both paper and electronic records are used by 47%. Only 2% of facilities assessed reported using electronic records as their sole source of patient record keeping. The most common electronic medical records utilized are Smartcare (75%) and DHIS2 (37%). Regular reporting of surgical outcomes to the health ministry is practiced by 84% of facilities including postoperative mortality (88%), maternal mortality (95%), and surgical complications (76%).

### 3.5 Research

Operating theater staff and OB/GYN's report the most ongoing research projects at 41% and 40% of facilities, respectively. Thirty‐two percent of facilities have ongoing general surgery‐led research projects, 31% of facilities have ongoing anesthesia‐led research projects, and 18% of facilities have ongoing orthopedic‐led research projects. The majority of research is observational (38%) and is only being conducted occasionally (65%). Health systems research (25%) and clinical trials (23%) are also reported.

### 3.6 Financing and Governance

Seventy‐two percent of healthcare facilities report a dedicated budget line for perioperative care. However, only 30% report an adequate budget with common challenges in funding related to insufficient allocations, competing needs, and a high demand for resources.

Clinical governance structures for perioperative services are in place at most facilities. Eighty‐two percent of facilities report having a surgical department. Although 40% report having a local committee dedicated to perioperative care, only 42% of committees were reported to be “effective” or “very effective.” The most common needs for governance support identified were training (81%), financial resources (67%), and policy support (44%). Only 43% of health facilities were aware of the existing NSOAP (2017–2021).

## 4 Discussion

ZAMSAT successfully captured surgical healthcare capacity data to inform the revision of the Republic of Zambia's NSOAP. With multistakeholder engagement, including the CSHR, WHO–Zambia, professional society leaders, ZOTNIG, and the MOH, data from 116 hospitals in all 10 provinces are reported.

ZAMSAT findings reveal both strengths and gaps in the delivery of surgical care and add to the limited available literature on the status of the Republic of Zambia's perioperative capacities and needs. Although progress is being made with the uptake of the WHO Surgical Safety Checklist usage and the use of pulse‐oximeters in theater, important deficiencies persist. One of the most striking findings is the lack of uninterrupted infrastructure components among facilities including clean water and electricity. This presents an opportunity for new collaborations with partners working to improve renewable energy and water, sanitation, and hygiene (WASH) for health facilities. Another notable finding was the gap in service delivery with only 37% of leveled facilities capable of open fracture management. This aligns with the findings from the NSHP and highlights the need for prioritizing continuous medical education for clinicians in open fracture management [18]. Although there is ongoing research at many facilities, most projects are observational and there is a gap in database availability to carry out more in‐depth studies. Only 2% of facilities are using electronic medical records which have been shown to improve research capacity and patient care [20]. With 80% of facilities reporting a surgical department and 40% reporting committees dedicated to perioperative care, ZAMSAT data reveal that the effectiveness of these establishments is limited. Additionally, less than half of all facilities were aware of the NSOAP underlining the need for improved data and policy dissemination.

The previous surgical capacity assessment, by Cheelo et al. in 2016, captured data on 39 hospitals. Their evaluation revealed 62% of hospitals had instruments for surgery that were always available and 62% had adequate drugs and consumables for theater care [9]. Our findings were different with only 21% percent of facilities reporting equipment and supplies always available for perioperative care. Some reasons for the difference in findings may be due to time elapsed, limited number of hospitals interviewed in 2016, methods of data collection and reporting, or differences in the facilities captured.

Although not a direct aim of ZAMSAT, the lack of ambulances provides insight into the gaps in interfacility transport. Only 52% of facilities report consistent access to ambulances and many surgical patients are waiting for transfer to higher levels of care due to fuel and working ambulance shortages. Although a focus needs to be placed on the improved provision of interfacility transport, there also needs to be a focus on bolstering Level 1 hospitals to decrease transport needs. Scaling up perioperative care at Level 1 hospitals has been identified as one solution to improving the outcomes of surgical patients [21, 22].

Zambia has a scarcity of trained surgeons and anesthesiologists in practice with the majority of perioperative care being performed by general doctors and anesthesia providers. This is not unlike other LMICs [23]. ZAMSAT did not capture the number of surgeons per 100,000 persons, but Zambia's NSHP estimates 0.6 general surgeons per 100,000 persons [18]. This is in sharp contrast to the recommendations from LCoGS of 6 general surgeons per 100,000 persons [1]. In a country where task‐sharing is accepted, there is an overwhelming need to provide more training opportunities for improving surgical skills in nonspecialists and nonphysician clinicians. To date, these opportunities are currently limited by few training sites and available training positions.

### 4.1 Next Steps

This paper describes the collaborative effort to rapidly assess baseline surgical capacity data at the national level to inform the NSOAP revision. The next steps are underway and the first NSOAP revision stakeholder meeting has been completed. The information provided by ZAMSAT was reviewed by key stakeholders including healthcare professionals, government representatives, nongovernmental organizations (NGOs), development partners, and patient representatives. Ongoing policy revision, writing, and validation are scheduled to be completed in 2025. To follow Zambia's NSOAP revision, three district‐level hospitals have been selected for pilot implementation.

### 4.2 Strengths

The strengths of this study include the diverse representation of facilities by level, location, and ownership type. Data from all 10 provinces were represented and facilities from all levels and rural and urban locations were included. Additional strengths include the rapid provision of usable data without large financial and human resources needed for collection. This project also provided information for stakeholders from the medical field, community, and government to identify priority growth areas for the scaling up of safe surgery in Zambia.

### 4.3 Limitations

With limited resources to complete the baseline assessment data collection, ZAMSAT did not allow for the collection of detailed information. The quantities of essential medication and equipment were not collected. Survey answers were also self‐reported. Additionally, we were not able to control for overlapping catchment populations and could not evaluate the distances and times to reach surgical care for Zambia's population. Specific data on surgical volumes were not collected due to limitations in how operative cases are recorded within facilities. For example, the general surgery theater logbook at the largest referral center in Zambia has been partially destroyed preventing laparotomy data collection. Zambia has placed a high priority on digitalizing healthcare as part of the revised NSOAP to fill some of these data gaps. Private hospitals are also underrepresented in our results. There is great value in using consistent surveying methods over time to accurately track progress and this survey method may not be transferable to other countries with other methods already in place. Lastly, we do not know the reasons why some hospitals did not complete the survey. Some surveys may not have been successfully distributed or returned due to internet connectivity issues. Despite these limitations, our findings represented an overall picture of the state of Zambia's surgical capacity and provided useful information that effectively informed the revision of Zambia's NSOAP.

## 5 Conclusion

The value of this study is the summary of the Republic of Zambia's perioperative capacity to inform the NSOAP revision. The rapid assessment's results will be useful for policy makers, medical providers, and donors when creating local initiatives to improve perioperative care in Zambia.

## Author Contributions

**Joshua Gazzetta:** conceptualization, methodology, project administration, writing – original draft. **Emmanuel Malabo Makasa:** conceptualization, investigation, project administration, supervision, writing – review and editing. **Moses Mwale:** data curation, formal analysis, methodology. **Cyrus Phiri:** investigation, project administration, writing – review and editing. **Mwamba Josephine Chiteba Mulenga:** investigation, project administration, writing – review and editing. **Mutimba Bernard Mpabalwani:** investigation, project administration, writing – review and editing. **Lillian Mwape:** investigation, project administration, validation. **Kennedy Lishimpi:** investigation, methodology, supervision.

## Conflicts of Interest

The authors declare no conflicts of interest.

## Supporting information

Supporting Information S1
