# Supplementary material for: Assessing the Perioperative Capacity in the Republic of Zambia in Preparation for the First Revision of the National Surgical Obstetric and Anesthesia Plan (NSOAP): A Rapid Survey Method
Source: World J Surg. 2025 May 6;49(6):1484–9. doi: 10.1002/wjs.12615 (PMC12134188; doi:10.1002/wjs.12615)
Supplement: Supplementary file 1 — Supporting Information S1 [file WJS-49-1484-s001.docx]

**THE EMERGENCY AND ESSENTIAL SURGICAL [PERI-OPERATIVE] HEALTHCARE**

**HEALTH FACILITY ASSESSMENT TOOL**

**GENERAL INFORMATION**

**Date of data collection (dd/mm/yy):** ______________________________________________________

**Name of Health Facility:_______________ Province: _____________ District:__________________**

**Level of your health facility:**  District (1)  Provincial (2)  Central (3)  Teaching (3)

 Other: please specify _______________________________________

**Facility Ownership Type:**   Public  Private  Mission/non-profit

 Other: please specify _______________________________________

**What is the population served by your health facility**: _______________________________________

**Respondents Name: _________________________ Position/Title: ____________________________**

**Phone #: ___________________________________ Email: _**__________________________________

**INFRASTRUCTURE**

1. **Does lack of electricity ever interrupt perioperative care?** Yes ☐ No ☐
2. **How often do interruptions in electricity supply occur?**
   - Never
   - Rarely (1-2 times a year)
   - Occasionally (3-6 times a year)
   - Frequently (more than 6 times a year)
3. **How long do these interruptions in electricity supply typically last?**

- Less than 1 hour
- 1-3 hours
- 3-6 hours
- More than 6 hours

1. **Does lack of clean water ever interrupt perioperative care?** Yes ☐ No ☐
2. **How often do interruptions in clean water supply occur?**

- Never
- Rarely (1-2 times a year)
- Occasionally (3-6 times a year)
- Frequently (more than 6 times a year)

1. **When interruptions in clean water supply occur, how do you manage perioperative care?**

- Use of stored water supplies
- Postpone surgeries
- Transfer patients to other facilities
- Other (please specify): [Text input]

1. **Does lack of oxygen ever interrupt perioperative care?** Yes ☐ No ☐
2. **How often do interruptions in oxygen supply occur?**

- Never
- Rarely (1-2 times a year)
- Occasionally (3-6 times a year)
- Frequently (more than 6 times a year)

1. **Does lack of internet ever interrupt perioperative care?** Yes ☐ No ☐
2. **Does lack of ambulance services ever interrupt perioperative care?** Yes ☐ No ☐
3. **When interruptions in ambulance services occur, how do you manage perioperative care?**

- Use of alternative transport
- Delay in patient transfers
- Other (please specify): ____________

1. **What is the impact of ambulance service interruptions on patient outcomes?**

- No impact
- Minor delays
- Major delays
- Increased complications
- Cancellation of procedures

1. **How many functional operating rooms (ORs) do you have?** *(Must be always working)________*
2. **How many ICU beds do you have?** *(Must be always working)________*
3. **How many PACU beds do you have?** *(Must be always working)_______*
4. **How many functional ventilators do you have?** *(Must be always working)_________*
5. **Is there a system in place for regular maintenance of surgical infrastructure and equipment?** Yes ☐ No ☐

**SERVICE DELIVERY**

1. **Are you always able to perform emergency laparotomy?** Yes ☐ No ☐
2. **Number of emergency laparotomies performed in the last 12 months:**_______
3. **Are you always able to perform caesarean sections?** Yes ☐ No ☐
4. **Number of caesarean sections performed in the last 12 months:** _________
5. **Are you always able to perform open fracture repairs?** Yes ☐ No ☐
6. **Number of open fracture repairs performed in the last 12 months: ___________**
7. **If you are not always able to perform Bellwether procedures, what are the reasons?**

- Lack of equipment
- Lack of trained staff
- Lack of supplies
- Other (please specify): _______

1. **Are surgical care services available 24/7?** Yes ☐ No ☐

**Quality and Safety**

1. **Do you always use the WHO surgical safety checklist?** Yes ☐ No ☐
2. **If you do not always use the WHO surgical safety checklist, what are the reasons?**

- Checklist not available
- Lack of training
- Time constraints
- Other (please specify): _________

1. **Do you always use pulse oximetry in the OR?** Yes ☐ No ☐
2. **If you do not always use pulse oximetry in the OR, what are the reasons?**

- Equipment not available
- Equipment malfunctioning
- Lack of training
- Other (please specify): __________

**Equipment and Supplies**

1. **Does the lack of equipment and supplies ever interrupt perioperative care?** Yes ☐ No ☐
2. **Is there a list of standardized equipment and supplies?** Yes ☐ No ☐
3. **Are there audits performed on the equipment and supplies (yearly at minimum)?** Yes ☐ No ☐
4. **How often is specific equipment or supplies unavailable?**

- Never
- Rarely (1-2 times a year)
- Occasionally (3-6 times a year)
- Frequently (more than 6 times a year)

1. **What is the impact on surgical outcomes when equipment or supplies are unavailable?**

- Minor delays
- Major delays
- Cancellation of procedures
- Increased complications

1. **Does lack of anaesthetic/paralytic drugs ever interrupt perioperative care?** Yes ☐ No ☐
2. **Does lack of sedatives ever interrupt perioperative care?** Yes ☐ No ☐
3. **Does lack of antibiotics ever interrupt perioperative care?** Yes ☐ No ☐
4. **Does lack of vasopressors ever interrupt perioperative care?** Yes ☐ No ☐
5. **Does lack of IV oxytocics ever interrupt perioperative care?** Yes ☐ No ☐
6. **Does lack of IV fluids ever interrupt perioperative care?** Yes ☐ No ☐
7. **Does lack of blood products ever interrupt perioperative care?** Yes ☐ No ☐
8. **Does lack of lab analysis ever interrupt perioperative care?** Yes ☐ No ☐
9. **Does lack of urinalysis ever interrupt perioperative care?** Yes ☐ No ☐
10. **Does lack of histopathology ever interrupt perioperative care?** Yes ☐ No ☐
11. **Do you have a functional X-ray?** Yes ☐ No ☐
12. **Do you have a functional ultrasound?** Yes ☐ No ☐
13. **Do you have a functional CT scanner?** Yes ☐ No ☐
14. **Do you have a functional MRI scanner?** Yes ☐ No ☐
15. **Do you have functional mammography?** Yes ☐ No ☐
16. **Do you have cervical cancer screening?** Yes ☐ No ☐
17. **Are internal skill development programs provided at your facility for surgical, obstetric, or anaesthesia (SAO) providers (at least yearly)?** Yes ☐ No ☐
18. **Are skills training provided by an outreach organization at your facility for surgical, obstetric, or anaesthesia (SAO) providers (at least yearly)?** Yes ☐ No ☐

**WORKFORCE**

1. **Are providers of surgery, obstetrics, and anaesthesia (SAO) available 24/7?**

- General Surgeon: Yes ☐ No ☐
- Orthopaedic Surgeon: Yes ☐ No ☐
- Anaesthesia provider: Yes ☐ No ☐
- OB/GYN: Yes ☐ No ☐
- General Doctor able to perform laparotomy, C-section, open fracture repair: Yes ☐ No ☐

1. **How many providers are available per shift?**

- 1-2
- 3-4
- 5 or more

1. **Are there any gaps in coverage, especially in emergencies?** Yes ☐ No ☐

- If yes, please specify: ____________

1. **Are the following subspecialties always available?**

- Neurosurgery: Yes ☐ No ☐
- Urology: Yes ☐ No ☐
- Maxillofacial: Yes ☐ No ☐
- Other (please specify): ____________

**INFORMATION MANAGEMENT**

1. **How do you keep patient medical records?**

- Paper
- Electronic
- Both

1. **Which electronic MHIN is in use at your facility? (Select all that apply)**

- DHIS2
- EIDSR
- DISA
- Smartcare
- Other Please specify___________

1. **How reliable are your electronic records systems?**

- Very reliable
- Reliable
- Unreliable
- Very unreliable

1. **Do you report the following to the government through the HMIS?**

- Postoperative Mortality: Yes ☐ No ☐
- Maternal Mortality: Yes ☐ No ☐
- Surgical Complications (e.g., Infections): Yes ☐ No ☐

1. **Are surgical outcomes (including postoperative mortality rates) regularly reported to the health ministry?** Yes ☐ No ☐

**RESEARCH**

1. **Do the following departments have ongoing research?**

- General surgery: Yes ☐ No ☐
- Orthopaedic surgery: Yes ☐ No ☐
- OB/GYN: Yes ☐ No ☐
- Anaesthesia: Yes ☐ No ☐
- Operating Theatre Nurses/staff: Yes ☐ No ☐

1. **What type of research is being conducted?**

- Clinical trials
- Observational studies
- Health systems research
- Other (please specify): _____________

1. **How often are research activities conducted?**

- Continuously
- Annually
- Biannually
- Occasionally

**FINANCING**

1. **Do you have a budget line specific for perioperative/surgical care?** Yes ☐ No ☐
2. **Is the budget for perioperative/surgical care adequate?** Yes ☐ No ☐

- If no, what are the challenges faced in securing funding? ___________________

**GOVERNANCE**

1. **Do your facility have a surgical department?** Yes ☐ No ☐
2. **Is there a local committee/working group dedicated to perioperative care?** Yes ☐ No ☐
3. **Was your facility aware of the National Surgical, Obstetric, and Anaesthesia Plan (NSOAP, 2017-2022)?** Yes ☐ No ☐
4. **How effective are these committees in addressing perioperative care issues?**

- Very effective
- Effective
- Ineffective
- Very ineffective

1. **What support is needed to strengthen governance structures?**

- Training
- Financial resources
- Policy support
- Other (please specify): _______________
